# Relationships between resting-state EEG functional networks organization and individual differences in mind wandering

**DOI:** 10.1038/s41598-022-25851-6

**Published:** 2022-12-08

**Authors:** Paweł Krukow, Kamil Jonak

**Affiliations:** 1 Department of Clinical Neuropsychiatry, Medical University of Lublin, Lublin, Poland; 2 Department of Computer Science, Lublin University of Technology, Lublin, Poland

**Keywords:** Imaging, Neuroscience, Physiology, Psychology, Risk factors

## Abstract

When performing cognitively demanding tasks, people tend to experience momentary distractions or personal associations that intercept their stream of consciousness. This phenomenon is known as Mind Wandering (MW) and it has become a subject of neuroscientific investigations. Off-task thoughts can be analyzed during task performance, but currently, MW is also understood as a dimension of individual differences in cognitive processing. We wanted to recognize the intrinsically-organized functional networks that could be considered the neuronal basis for MW dispositional variability. To achieve this goal we recruited a group of normal adults, and eventually divided the group in half, based on participants’ scores on the scale measuring dispositional MW. Next, these groups were compared regarding the arrangement of preselected intrinsic functional networks, which were reconstructed based on multi-channel signal-source resting-state EEG. It appeared that subjects who tend to mind wander often exhibited decreased synchronization within the default mode network, and, simultaneously, strengthened connectivity between ‘on-task’ networks of diverse functional specificity. Such within- and between networks integrity patterns might suggest that greater Mind Wanderers present an atypical organization of resting-state brain activity, which may translate into attenuated resources needed to maintain attentional control in task-related conditions.

## Introduction

Over the last two decades, there has been a considerable shift in the interest in cognitive neuroscience, from the analysis focused exclusively on modularly specific cognitive functions (e.g. working memory) and their neuronal underpinnings, towards more spontaneously revealing, task-unrelated mental processes. This shift can be explained by the finding that resting-state brain activity is strictly organized and cannot be treated in terms of random neural noise^1^, as well as by psychological studies confirming that a significant range of mental activity is not focused on any specific task, and yet, it engages at least 30% of our time^2^. Among various types of spontaneously-generated thoughts, one can distinguish mind wandering (MW), also called “off-task thoughts” or task-unrelated thoughts defined as a shift of attention away from the primary task inwards^3–5^. Associations between MW and task performance are expressed in a form of methods used in a substantial amount of previously conducted MW-studies. For example, Gruberger et al.^6^ in their review regarding neuroscientific research on MW indicate that the most common solution was the application of the so-called "thought probes", when subjects are explicitly asked whether their current thoughts were focused on the task or not, during the performance. Another strategy consists in experimental manipulation of cognitive load, resulting from the assumption that tasks with a minimum level of complexity or other feature enabling its effortless performance increase the range of MW.

It is postulated that spontaneous thoughts and daydreaming play an important role in integrating the self-referential processes, increasing the level of autobiographical memory consistency and improving creativity and future-oriented planning^7,8^, thus, these experiences might have an adaptive psychological function. On the other hand, if one considers MW as a state of unintentional detachment of attention from the task, then the consequences for the task being performed will be rather detrimental^4,9^. When MW is understood in the context of mental activity control failures, research indicates its relationship with less adaptive features or states of reduced cognitive efficiency. Individuals exhibiting more pronounced tendency to mind wander obtain higher scores in scales measuring dispositional neuroticism^10^, negative affect and lower levels of effortful control and extraversion^11^. Enhanced MW was observed during states of reduced alertness, fatigue and generally diminished productivity^12,13^. Moreover, augmented propensity to MW might be considered as a risk factor for mental health, having in mind its significant relationships with negative affect and anxiety^2,14,15^.

The self-relatedness of spontaneous thoughts clearly suggests its associations with the activity of neural areas constituting the Default Mode Network (DMN, a set of neuronal regions activating in a synchronized manner during rest and redirection of attention on internal experiences, 16). A vast amount of fMRI research confirms that during the off-task state, the most pronounced neural activity can be observed mainly within the DMN regions, such as areas located within the brain's medial wall (e.g., medial prefrontal cortex, anterior and posterior cingulate cortex, precuneus), together with parahippocampal structures^16,17^. However, further studies revealed that associations between neural networks and MW are not only limited to the activity of the DMN, but also exhibit connectedness with much more complex inter-networks relationships. WM turned out to be associated with the strength of anti-correlations between sets of synchronized areas activated while performing a task (so-called task-on networks) and collection of regions whose activity is more pronounced during rest (so-called task-off networks) and even with the involvement of a set of neuronal structures engaged in and motor performance^18–20^. Participation of non-DMN areas in the states of MW was also confirmed by the results of the meta-analysis by Fox et al.^17^ on functional neuroimaging studies on spontaneous thoughts including MW. The authors underlined that beside recurring findings confirming relationships between task-off mentations and DMN, the engagement of the frontoparietal network, secondary somatosensory cortex, insula, lingual gyrus and temporopolar cortex, were also noted. The meta-analysis also highlights the role of inter-networks integration and overlapping activity co-occurring within structures from default and frontoparietal control networks. It should be also emphasized that the results regarding the strength of within-DMN activity and functional connectivity (FC) and MW proneness, are also not entirely unambiguous, e.g. Poeiro with co-workers^21^ found that intensified internal engagement of attention was linked to stronger coupling between DMN subsystems, while Groot et al.^22^ using combined fMRI and EEG recordings obtained results suggesting weaker within-DMN activity associated with task-unrelated thoughts. In other words, regardless of the undisputed progress in research on the neural signature of MW, one cannot conclude that the neurophysiological basis of this phenomenon is fully understood.

Although the majority of studies on MW used methodological solutions referring to this phenomenon as ongoing during the performance of experimental task, there is an increasing amount of research suggesting that MW might be also treated as a dimension of individual differences in cognitive processing, in other words, as a trait^23,24^. One of the important methodological aspects enabling research on dispositional MW was the introduction of questionnaire methods enabling precise and accurate MW assessment. Taking into account the narrow definition of MW in which the negative influence of MW on the task performance is underlined, Mrazek et al.^25^ developed a short self-report scale (Mind Wandering Questionnaire, MWQ) measuring the frequency with which an unintentional redirection of attention from the main task inwards occurs. There are also some initial neuroscientific studies regarding associations between dispositional MW and brain activity, usually analyzed with reference to the functional neural networks organization, which might be explained by the high amount of previous experimental outcomes linking MW with DMN^26^. This research documented that the variability in the resting-state functional networks configuration is a significant basis for individual differences in cognitive functioning, also when both MW and neural activity are not analyzed with the reference to the actual task being performed. Even more so, it seems justified that the dispositional MW variability is associated with important dimensions of the brain's organization.

Considering the above, the goal of our study was to broaden existing knowledge regarding functional neural networks’ contribution to individual differences in MW, however, we would like to propose some different methodological and technical solutions compared with the previous studies. In detail, we wanted to determine, whether individual differences regarding MW present in the sample of healthy young adults, might be reflected by variance in the organization of preselected, resting-state neural networks reconstructed on the basis of source-space multichannel EEG. Compared with fMRI-based FC relying on the correlations between co-occurring BOLD signal low-frequency fluctuations^27^, EEG-derived connectivity is computed from temporal synchronization between selected features of the electrophysiological signals informative of neuronal oscillators acting within narrow frequency windows^28^. Modelling of the functional network arrangement based on the EEG might be additionally improved by applying methods implementing electrophysiological signal source reconstruction, which is particularly important in networks studies as it enables more precise neuroanatomical localization of signals’ neuronal origins, and solves the problem of volume conduction burdening some computational approaches utilized in EEG signal-based analyses^29^.

## Results

### Characteristics of the studied group and its division based on the MWQ results

**Figure 1:**
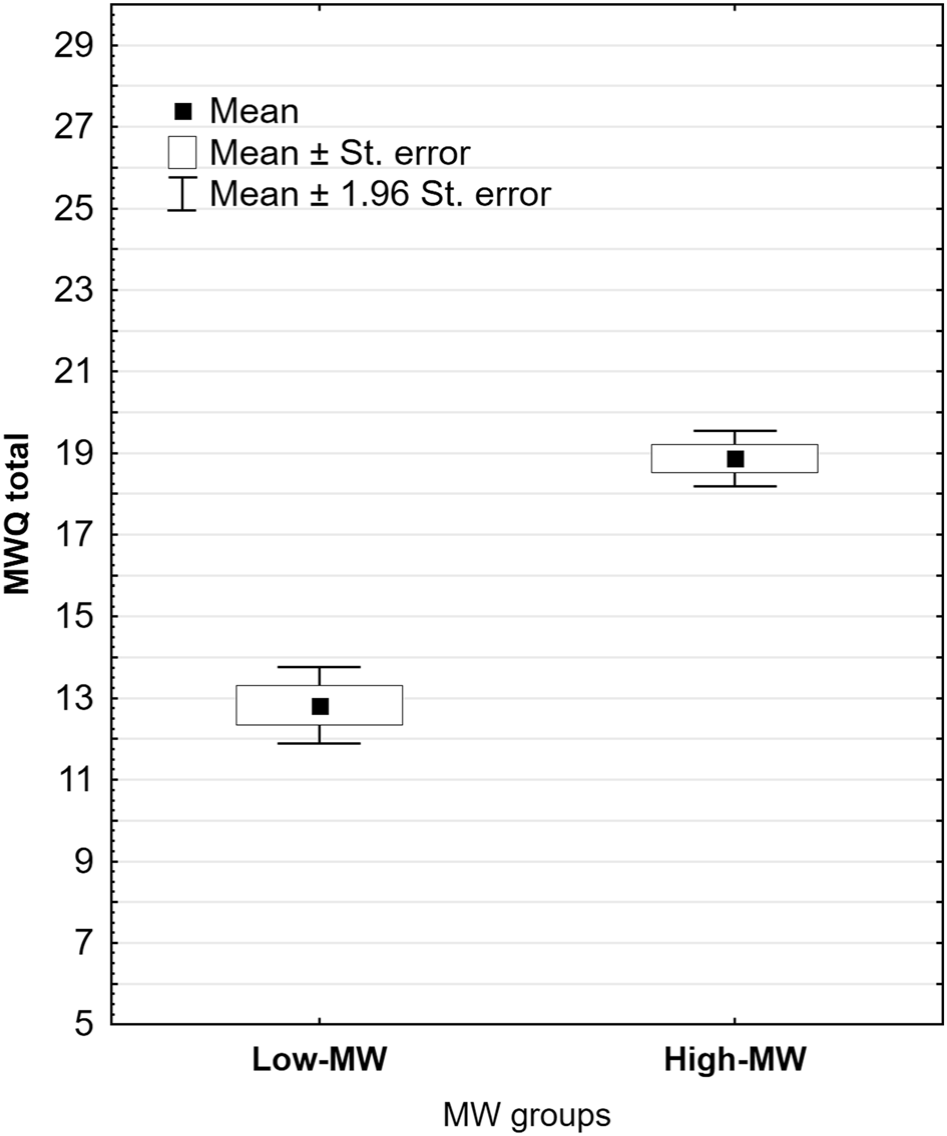
The box plot showing the range of the difference in the total Mind Wandering Questionnaire (WMQ) score present in two empirically established groups. Note. st. error: standard error of measurement.

After applying all mention inclusion and exclusion criteria and reviewing EEG recordings the final sample which outcomes were analyzed contained 66 participants with mean age of 22.66 (SD = 2.46), 15.33 (SD = 1.66) years of education, and 90.46 (SD = 2.86) Percentile Ranks (PR) obtained in Raven’s Standard Progressive Matrices (SPM) test. The subgroups of students from a technical and a medical university did not differ in terms of variables such as age, gender, and years of education (all p < 0.2). In the whole group, there were 36 females and 30 males, all Caucasian. Mean MWQ was 16.22 (SD = 3.64; min. = 6, max. = 24), and the median 17 points. MWQ distribution did not deviate from normal. According to MWQ median, the whole sample was divided into two subgroups, one with MWQ results ranging from 6 to 16 (Low-MW group) and the second one with MWQ results ranging from 17 to 24 points (High-MW group). Figure 1. presents a box plot of the total MWQ score in these samples. The range of scores possible to obtain in MWQ ranged from 5 to 30 points. Two empirically established groups were similar with regard to age, sex, education and fluid intelligence (Table 1).

**Table 1:** Demographic and cognitive characteristics of two empirically-distinguished groups differing with regard to the level of dispositional MW.

	High-MW group (n = 34)	Low-MW group (n = 32)	t/χ^2^	p	Cohen’s d/r
Age	21.25 (1.59)	21.72 (2.81)	0.792	0.429	0.20
Sex (% male)	41	50	2.133	0.144	0.17
Years of education	14.96 (1.86)	15.48 (2.06)	0.882	0.224	0.26
SPM (PR)	90.19 (2.54)	90.65 (2.66)	0.686	0.495	0.17
MWQ	18.87 (1.92)	12.82 (2.59)	10.871	< 0.0001	2.65

High-MW: a subgroup with higher MWQ total score, Low-MW: a subgroup with lower MWQ total score.

### Networks connectivity organization in groups with low and high MW levels

Since there were no significant between-group differences in age, sex, education and fluid intelligence, PLV indicators of functional connectivity strength for preselected pairs of nodes has been compared in two groups differing with regard to dispositional Mind Wandering. According to the scheme of statistical analysis described in the Methods section, the first step aimed at identifying potential differences regarding intra-network connectivity strength. In this analysis the permutation statistics from the Resampling Statistical Toolkit included 785 tests, 35 between-group differences appeared to be significant, after inclusion of the false discovery rate (FDR) correction 18 of them remained statistically significant.

**Figure 2:**
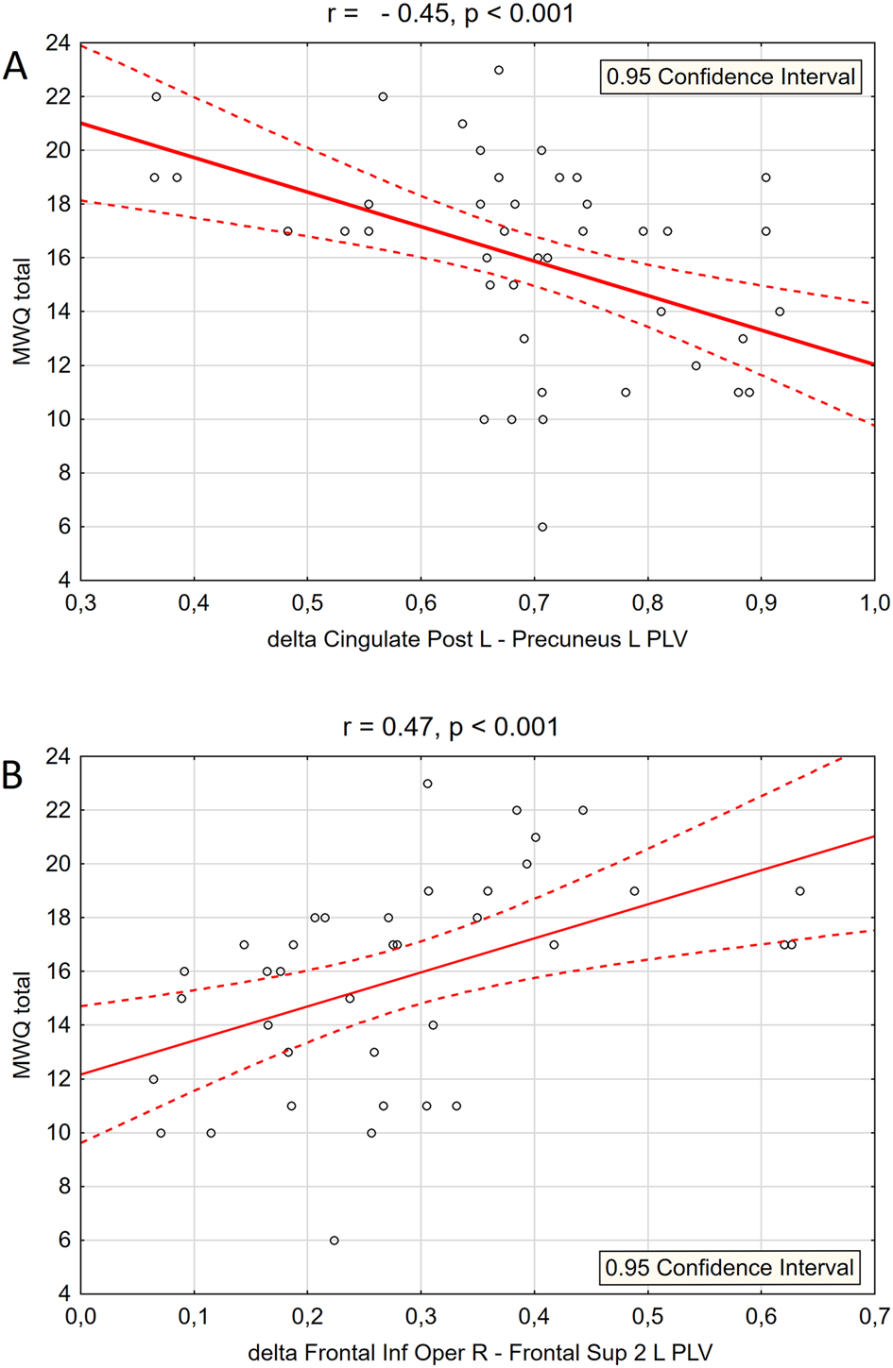
Two scatterplots showing linear associations between the total score obtained by all participants in the MWQ and selected PLV values: (**A**) negative correlation between MWQ and intra-DMN connectivity, (**B**) positive correlations between MWQ and inter-networks connectivity covering structures belonging to the CON and FPN network in delta band.

**Figure 3:**
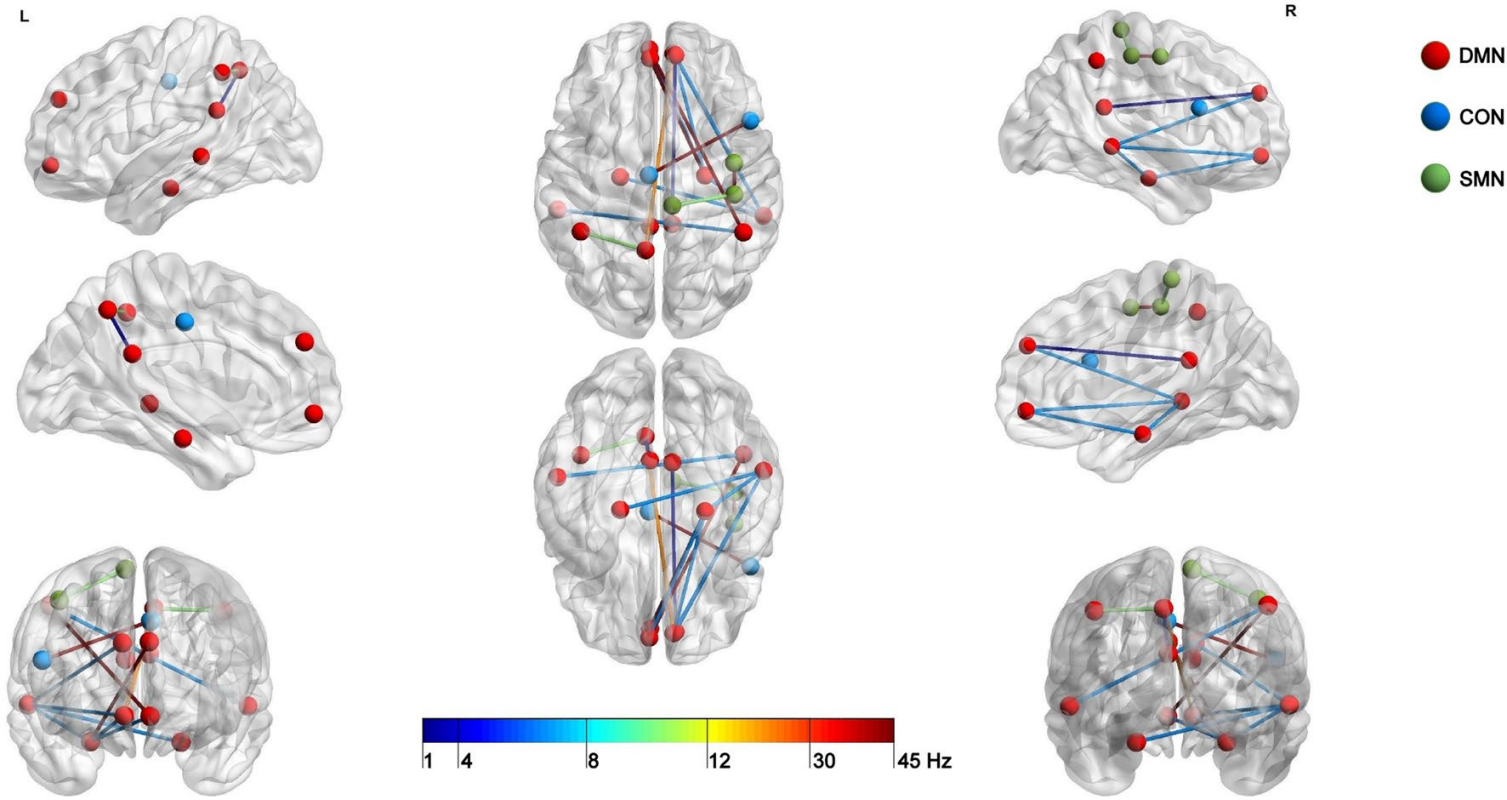
Schematic localization of nodes and intra-network functional connections, which measures significantly differentiated groups categorized on the basis of dispositional Mind Wandering. Nodes are indicated as balls and connections as lines. Red nodes belong to DMN, blue to CON, and green to SMN. According to added scale, line colors represent frequency bands. Dark and light blue lines indicate connections in delta and theta bands, light green alpha, yellow beta and red gamma bands.

Table 2 contains results of this comparison. Among 18 FC-indicators significantly differentiated the groups, 14 included ROIs located within DMN, 3 were localized within Sensory-Motor Network (SMN) and one within Cingulo-Opercular Network (CON). All PLV values differentiating groups in lower frequencies (delta and theta) covered within-DMN regions, and in all these cases within-DMN connectivity strength was lower in High-MW group comparing with Low-MW group. On the other hand, all 3 measures of the within-SMN connectivity strength was significantly higher in the High-WM sample than in the Low-MW group. The strength of gamma-band connectivity between left middle cingulate gyrus and the right frontal inferior operculum, both belonging to the CON was also higher in High-WM group than in Low-MW group. The majority of these ROIs were located in the right hemisphere (23 *versus* 13 located in the left). Significant relationships between MWQ and FC has been additionally confirmed by correlation analyses. Figure 2 shows linear relationships between MWQ total score and selected PLV values in the whole research sample. Figure 3. shows the schematic localization of synchronized nodes, which connectivity strength significantly differentiated subpopulations with low and high level of dispositional Mind Wandering.

**Table 2:** Intra-network differences between subpopulations differing with regard to dispositional Mind Wandering and the results of Pearson’s linear correlations (r) between MWQ total score and functional connectivity values measured by the PLV in the whole sample.

Frequency	Functional network	Synchronized pair of nodes	High-MW PLV	Low-MW PLV	p_corr_	MWQ-PLVr
Delta	DMN	Cingulate_Post L—Precuneus L	0.64	0.76	0.044	− 0.45***
Delta	DMN	Cingulate_Post R—Frontal_Sup_Medial R	0.24	0.39	0.044	− 0.36**
Theta	DMN	Frontal_Med_Orb R—ParaHippocampal R	0.23	0.39	0.044	− 0.36**
Theta	DMN	Frontal_Med_Orb R—Temporal_Mid R	0.21	0.39	0.044	− 0.34**
Theta	DMN	Frontal_Sup_Medial L—Temporal_Mid R	0.22	0.35	0.044	− 0.35**
Theta	DMN	Parietal_Inf R—Temporal_Mid L	0.19	0.31	0.044	− 0.37**
Theta	DMN	Frontal_Med_Orb L—ParaHippocampal R	0.36	0.51	0.049	− 0.40**
Theta	DMN	ParaHippocampal L—Temporal_Mid R	0.24	0.38	0.049	− 0.20
Theta	DMN	ParaHippocampal R—Temporal_Mid R	0.52	0.67	0.049	- 0.27*
Alpha	DMN	Parietal_Inf L—Precuneus L	0.67	0.50	0.049	0.36**
Alpha	SMN	Paracentral_Lobule R—Postcentral R	0.58	0.42	0.044	0.37**
Beta	DMN	Frontal_Med_Orb R—Precuneus L	0.25	0.16	0.044	0.41***
Beta	SMN	Postcentral R—Precentral R	0.85	0.78	0.049	0.33*
Gamma	CON	Cingulate_Mid L—Frontal_Inf_Oper R	0.25	0.13	0.044	0.41***
Gamma	SMN	Postcentral R—Precentral R	0.80	0.69	0.044	0.35**
Gamma	DMN	Frontal_Sup_Medial L—ParaHippocampal R	0.14	0.25	0.044	− 0.47***
Gamma	DMN	Frontal_Sup_Medial L—Temporal_Mid R	0.17	0.28	0.044	− 0.32*
Gamma	DMN	Frontal_Med_Orb L—Parietal_Inf R	0.19	0.12	0.049	0.28*

p_corr_—permutation test’ level of statistical significance, including FDR correction 23R, 13R.

*Correlation significant at p < 0.05.

**Correlation significant at p < 0.01.

***Correlation significant at p < 0.001.

In the next stage, the groups were compared in terms of the strength of synchronization between nodes located in different functional networks. Table 3. shows the statistically significant results of this comparison performed with the usage of the permutation test, and correlations between MWQ total score and selected PLV values in the whole sample.

**Table 3:** Inter-networks significant differences between subpopulations differing with regard to dispositional Mind Wandering and the results of Pearson’s linear correlations (r) between MWQ total score and PLV in the whole sample.

Frequency	Functional networks	Synchronized pair of nodes	High-MWPLV	Low-MWPLV	p_corr_	MWQ-PLVr
Delta	CON-FPN	Frontal_Inf_Oper R—Frontal_Sup_L	0.36	0.19	0.044	0.47***
Delta	CON-FPN	Frontal_Inf_Oper R—Frontal_Mid_ L	0.38	0.21	0.044	0.39**
Delta	SMN-DMN	Paracentral_Lobule L—Parietal_Inf L	0.21	0.39	0.044	- 0.36*
Delta	SN-DMN	Cingulate_Ant L—Cingulate_Post R	0.48	0.31	0.049	0.38**
Theta	CON-FPN	Frontal_Sup_L—Thalamus L	0.63	0.46	0.044	0.45***
Alpha	SMN-FPN	Paracentral_Lobule R—Parietal_Sup R	0.58	0.38	0.049	0.29
Alpha	DMN-CON	Frontal_Sup_Medial L—Rolandic_Oper L	0.31	0.19	0.049	0.47***
Alpha	CON-FPN	Frontal_Sup_R—Thalamus R	0.23	0.36	0.049	- 0.39*
Alpha	SMN-DMN	Paracentral_Lobule R—Parietal_Inf R	0.54	0.35	0.049	0.29
Beta	SMN-FPN	Frontal_Mid_R—Precentral L	0.31	0.19	0.049	0.37*

p_corr_—permutation test’ level of statistical significance, including FDR correction.

*Correlation significant at p < 0.05.

**Correlation significant at p < 0.01.

***Correlation significant at p < 0.001.

**Figure 4:**
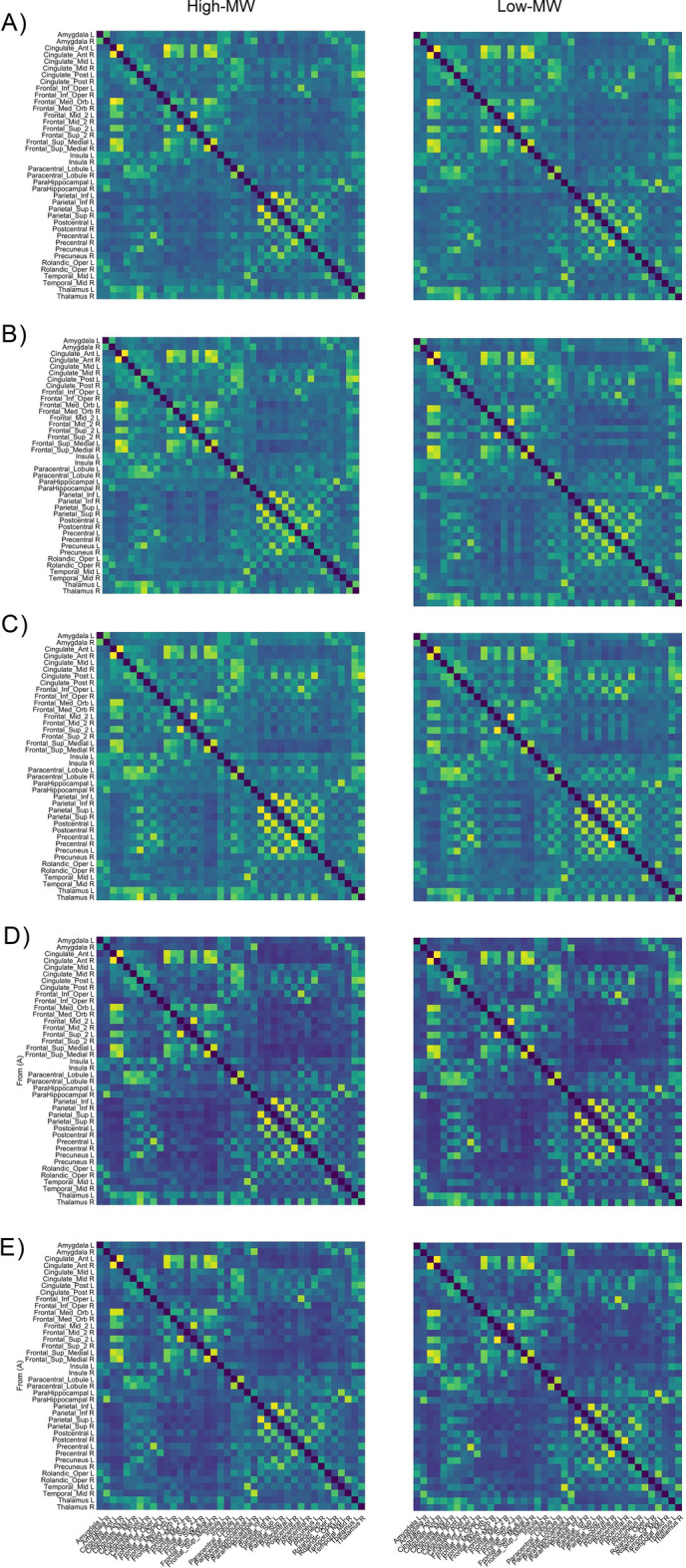
Connectivity matrices covering all 40 included nodes, representing the distribution of synchronizations strength in (**A**) delta, (**B**) theta, (**C**) alpha, (**D**) beta and (**E**) gamma bands separately for groups differing regarding the level of dispositional Mind Wandering. Matrices of the High-MW group are on the left, and the Low-MW on the right.

Inter-networks analyses with the permutation statistics covered 3115 test, 156 between-group differences appeared to be significant, after inclusion of FDR correction 10 of them remained statistically significant. Between-network connectivity strength for eight pairs of nodes was higher in the High-WM sample compared with the Low-MW sample. Groups differentiating synchronizations involved pairs of structures connecting CON and Fronto-Parietal Network (FPN, four cases), SMN and FPN (two cases), SMN and DMN (two cases), DMN and CON (one case) and DMN and SN (one case). Figure 4 presents connectivity matrices covering included neuronal areas constituting preselected functional networks.

## Discussion

The goal of our study was to verify whether there are noticeable individual differences in the organization of intrinsic functional networks being the neural basis of variance in dispositional Mind Wandering. Additionally, we wanted to add some potentially new findings based on the EEG technique to the current scope of knowledge dominated by fMRI-derived outcomes. Reconstruction of neural networks based on signal-source EEG may produce different results than fMRI-derived data because the FC indicators measured by EEG and fMRI are based on at least partially different neurophysiological mechanisms and other computational procedures^30,31^.

Having the empirical distribution of the MWQ total score, we divided the group in half obtaining subgroups of participants identified as those exhibiting low and high trait-tendency to Mind Wander. These subgroups appeared to differ regarding the organization of the intrinsic functional networks. The differences considered within-network consistency and between-networks synchronizations. The subgroup of high mind-wanderers displayed pronounced under-connectivity within Default Mode Network (DMN). The diminished synchronization covered sets of structures forming main DMN hubs, like posterior cingulate and precuneus constituting the backside hub, but under-connectivity was observed also between posterior and anterior hubs and between these areas and structures of the medial temporal lobe with parahippocampal gyrus. Decreased within-DMN connectivity strength was present mainly in low frequencies (delta and theta), however individual FC indicators differentiating the groups occurred also in higher frequencies (beta, gamma), mainly between medial frontal and temporal areas. Besides reduced DMN integration, a subgroup with higher MWQ score displayed increased connectivity between neuronal areas belonging to Sensory-Motor Network (SMN, precentral—postcentral gyri) and Cingulo-Opercular Network (CON, middle cingulate—frontal inferior operculum). These synchronizations occurred mainly in higher frequencies. As for within-networks organization, there were no significant between-groups differences regarding Fronto-Parietal (FPN) and Salience networks (SN). The inter-network synchronization indicators also differentiated the subgroups, in this case those who mind-wander often exhibited stronger interconnectedness between regions belonging to the networks of different functional characteristics. Four out of ten pairs of connections included links between FPN and CON, the other interconnections that differentiated the groups embraced the SMN and DMN nodes. Relationships between individual differences in MW and the organization of intrinsic functional networks has been additionally confirmed by correlations analyses. Summarizing, it can be concluded that the majority of the obtained intergroup differences suggest that high mind-wanderers displayed weakened intra-network integration, primarily within the DMN, and simultaneously increased inter-network coordination, which mainly included synchronization of the frontal and somatosensory cortex with other structures. What seems to be of special importance here, these network organization patterns reflect neurophysiological activity recorded in resting-state condition. On the other hand, connectivity matrices shown in Fig. 4 suggest, that the structure of connectivity was rather similar than different, and that there were no radical differences between synchronizations arrangement in all five frequency bands. However, these resemblances might be explained by the fact that comparison involves two groups of demographically and similar healthy individuals.

When our outcomes are related to Gruberger et al.^6^ review on the neural determinants of Mind Wandering, it seems that our results are in line with the majority of previously reported findings linking MW with the DMN, as well as with the areas belonging to the so-called 'on-task' functional networks. At this level of generality, it can be also concluded that our outcomes are consistent with the Godwin et al. study^32^, in which authors likewise intended to determine the relationship between a trait-level MW and the organization of resting-state neural networks. However, a more in-depth analysis shows that our data is in some critical aspect different from Godwin’s findings. First of all, our results suggest, contrary to the above-mentioned ones, that intensified MW propensity is associated with intra-DMN underconnectivity, not increased connectivity. Additionally, we did not observed significant associations between dispositional MW and DMN-FPN synchronizations, more than half of the significant between-group differences related to inter-network connectivity considered elevated integration among on-task networks, e.g. CON-FPN, SMN-FPN. Dealing with some methodological solutions utilized in our study, as well as some part of obtained results, the present research differs from some previous neurophysiological investigations. Notably, we do not know of the published studies that searched for the relationship between MW understood as a trait and the organization of functional networks reconstructed on the basis of source-space resting-state EEG. Previous EEG studies aimed, inter alia, at establishing links between MW and the theta/beta ratio^33^, or generally a given frequency peak^34^, and identifying EEG signature of off-task though using thought-probe method and ERP analysis^35,36^. The frequency-focused investigations have produced quite divergent results suggesting that MW is associated with alpha^36,37^ delta and gamma^38^ or by delta, theta and alpha bands^39^. In this aspect, functional connections which differentiated our groups have occurred mainly in the theta, delta and gamma bands. The difference regarding frequencies may, on the one hand, result from the different methodological solutions used in our study compared to the previous ones, which often applied a though-probe methodology and task-related EEG recordings analyses, such as ERPs^35^. In addition, other authors narrowed down the range of the analyzed frequencies or examined only certain values of the proportion of low and high frequencies^33^. It should be also pointed out that some portion of past EEG research on MW did not carry out FC or network analysis at all, but used the time–frequency analyzes in a predefined frequency range instead^37^. On the other hand, neuroimaging studies on MW and its relations with the organization of functional networks were carried out almost exclusively with the use of fMRI^6,40^, and still, despite the plethora of evidence linking MW with DMN, the exact direction of these associations and its explanations can hardly be described as unequivocal^22,41^.

The fundamental difference between our outcomes showing MW—functional networks associations and the greater part of fMRI results suggesting that MW states are reflected by DMN increased connectivity might be explained by the fact, that the majority of fMRI investigations were carried on during task performance, which entails the activation of the on-task networks as a basic state, only occasionally interrupted by MW intrusions. Such off-task interference somehow must be underpinned by an increase in off-task network connectivity, because networks characterized by opposing functional characteristics are anti-correlated^1,42^. In purely resting-state paradigm, such experimentally-induced relationships between networks may not occur, therefore results of our study cannot be fully comparable with described fMRI surveys.

The current study, however, may shed some new light on the neural basis of MW individual differences. In resting-state condition, our subgroup with significantly higher scores in MW questionnaire displayed decreased connectivity within DMN and increased connectivity between some on-task networks. This arrangement seems to be almost exactly the opposite of the resting-state brain activity organization, on the contrary, it more closely resembles the configuration of neural networks activated by the performance of cognitively engaging tasks. In other words, mind wanderers may exhibit an unusual level of intrinsic readiness or alertness inadequate to the repose mode. If so, then a prolonged state of internally-induced alertness may eventually drain the resources needed to maintain attention control and this will result in more frequent attentional lapses in task-related situations. Perhaps future studies will corroborate this interpretation, taking into account the current data confirming the relationship between the resting-state neuronal networks interactions and the indicators of the autonomic system activity, which is also an important factor in mechanisms maintaining vigilance^43^. This notion is only theoretical speculation, although, relationships between DMN underconnectivity and attentional lapses were lately found in patients with attention-deficit/hyperactivity disorder^44^. Additionally, constantly intensified alertness might increase distractibility and this also may lead individuals to describe themselves as being less able to stay focused while performing a task. Our results are in line with Poole and co-workers^45^ findings, according to which decreased intra-DMN connectivity is significantly associated with poorer distractor suppression. Of note, Mohan et al.^46^ review regarding DMN connectivity in various neuropsychiatric conditions also suggests that this network resting hypo-synchronization was observed in selected neurodegenerative diseases characterized primarily by cognitive decline. These conclusions suggest that our results can be inscribed in the existing knowledge regarding the relationship between the coherence and organization of neural networks and relatively less favorable mode of information processing.

Lastly, we would like to mark that presented research has some limitations which should be addressed. Our study design did not relied on experimental variables manipulation, therefore, cause-and-effect interpretations should be cautious. A set of neuronal structures included in the network analysis has not contained all areas within the scope of neuroanatomical atlases used for localization of EEG signal sources, moreover, we did not included all possible types of functional networks. Limiting the nodes number was entirely deliberate, first of all, there is available literature showing which kind of networks should be reasonably included^40^, secondly it is necessary to constrain number of vast connections multiplication, what is typical for the EEG study, when the results are to be investigated in many frequencies. We used quite restrictive criteria for inclusion and exclusion from the study, some of them, e.g. lack of promotion to the next grade, is not among the criteria most often used in studies on the general population, but this criterion was included as an attempt to make participants similar. Our study does not decide about the psychological nature of MW, research and discussions on whether it is a phenomenon related to individual differences, cognitive or personality psychology continues and bring new results showing that MW is associated on the one hand with executive attention and on the other hand with neuroticism^47^. Considering such complex relationships, it seems reasonable to suggest that future studies on neuronal networks basis of MW should control for possible impact of personality traits and possible co-linearity between MW assessed with self-descriptive methods and neuroticism or a personality-conditioned tendency to over-concern. If mind wandering is primarily a reflection of a tendency to worry or an expression of diminished effectiveness of executive attention^48^, then its neural basis should be investigated in terms of the broader context of psychological variables.

## Methods

### Participants

As noted earlier, the study aimed to recruit healthy participants, so we invited 100 students, aged 21–24 years, males and females, representing various fields of study from two different universities with medical and technical profiles. The participants were informed about the purpose of the study and took part in it without financial reward. After the initial recruitment process, all individuals were screened regarding specific criteria. Based on the interview conducted by the psychologist, potentially excluding features were assessed, such as past experience of traumatic brain injuries, neurological and psychiatric diseases, taking any medications that may affect the EEG recording and cognitive functioning, psychoactive substances addictions, serious sleep problems, mental health problems requiring medical consultations, or taking medications prescribed by a psychiatrist also for the first-degree relatives of the study participants. In addition, the further excluding factors were left-handedness, learning difficulties diagnosed in the past in the form of neurodevelopmental disorders diagnosis and the lack of promotion to the next grade in primary or secondary school. After the described screening, the group number decreased to 84 individuals who had undergone further assessment consisting in the application of Raven’s Progressive Matrices test, Mind Wandering Questionnaire and resting-state EEG. All applied methods were carried out in accordance with relevant guidelines and regulations, all experimental protocols were approved by a Bioethics Committee of the Medical University of Lublin. Informed consent was obtained from all subjects.

### Evaluation of cognitive individual differences

To assess fluid intelligence a Raven’s Standard Progressive Matrices (SPM) test in a polish adaptation^49^ has been administrated. SPM consists of 60 tasks arranged in 5 series (A, B, C, D, E), 12 tasks each. The test material is in the form of incomplete formulas (matrices), and the respondent task is to select the missing fragment from the provided ones. This method is described as almost entirely culturally neutral, with non-verbal material and very simple instructions. The test primarily examines reasoning on visual material and is standardly used to assess individual differences in the level of intelligence dimension which minimally depend on education, and to a greater extent on innate factors^50^. The Polish version of the test has high psychometric parameters in terms of reliability (high internal stability) and validity, in the form of high correlations with other measures of general intelligence. To ensure a relatively high level of demographic and cognitive homogeneity in our sample, only individuals whose SPM's results range between 75 and 95 Percentile Rank (which corresponds to Wechsler-type IQs between 110 and 125^51^, has been incorporated into the final research group.

The Mind Wandering Questionnaire (MWQ)^25^ in polish translation was used to assess dispositional MW. MWQ is a single-factor short questionnaire containing 5 items openly referring to the theoretical construct of MW as an uncontrolled redirection of attentional resources from the ongoing task, e.g. “*I do things without paying full attention*”, „*I find myself listening with one ear, thinking about something else at the same time*”, “*I mind-wander during lectures or presentations*”. Responders answer on a 6-point Likert-type scale that goes from 1 (almost never) to 6 (almost always). The range of results extends from 5 to 30 points. So far, MWQ has been translated into other non-English languages^52,53^, all adaptations confirmed single-factorial structure and questionnaire good internal consistency. The authors of the questionnaire argued that the development of a new scale was necessary because previously existing methods such as the Daydream Frequency Scale (DDFS)^54^, or the Attention Related Cognitive Errors Scale (ARCES)^55^ did not sufficiently highlight the specificity of MW in the context of other types of spontaneous thoughts, did not have established face validity, and above all did not emphasize substantial relevance of the MW with the currently performed task.

Polish version^56^ also had satisfactory internal consistency, inter-item correlations reached between 0.40 and 0.62. Exploratory factor analysis also corroborated single-factorial structure of the scale, with eigenvalue reaching 2.641 and explained variance 52.8%, the Cronbach’s α was 0.78. Each of the five MWQ items had a high load towards an unique factor which might be called MW (0.67, 0.79, 0.70, 0.73, 0.81).

### EEG recording and preprocessing resting-state functional networks reconstruction

First, 15 min of resting-state EEG (eyes closed) data were recorded from all of the participants. EEG examinations were done in a well-lit and quiet room with the use of a 64-channel HydroCel Geodesic Sensor Net (Electrical Geodesics Incorporated, Eugene, OR, USA) with Ag–AgCl electrodes. During the data recording, the sampling rate was kept at 1 kHz with the application of a vertex reference using the NetStation 5.4. software package^57^. Impedances of the electrodes were maintained below 65 kW. Additionally, during the recording, a band-pass filter (0.5 to 70 Hz) and an active notch filter were used (50 Hz). Visual inspection of the spline interpolation of bad channels was made. After having been recorded, the NetStation software was used for the data conversion to the ASCII format.

In the next stage of the analysis, the EEG recordings in ASCII files format were imported to EEGLAB v.13.5.4b1 (http://sccn.ucsd.edu/eeglab/index.html), which is an open-source toolbox for MATLAB^58^. Then, the EEG signals were re-referenced to the common average reference and filtered with a bandpass Hamming window 0.5–45 Hz filter. After the filtering procedure, each EEG data set was segmented into 150 epochs, with a duration of 4096 samples (approximately 4 s) each. After the segmentation, each epoch was visually inspected by a certified clinical neurophysiologist, who removed bad epochs containing artifacts (i.e., head or muscle movements, electrode cable movements, or jaw clenching) from the analysis. Finally, for each participant, 135 visual-artifact-free epochs were selected.

To calculate the source activities from the recorded scalp EEG signals, a sLoreta implemented in the Brainstorm toolbox was used. The sLORETA^59^ algorithm allows for the analysis of EEG signals with zero localization error under noise-free conditions. These properties are ensured primarily by the signal; hence, the images obtained represent the current density and present the point of exact source localization. The ICBM152 MRI brain template and the deep brain structures provided by the Brainstorm toolbox were employed to estimate neuronal activities. The lead field matrix was constructed using a three-layer boundary element model provided by the OpenMEEG 2.4.1.^60^ project software. The specified regions of interest (ROIs) were selected from the AAL90 brain atlas. Among the 30,020 nodes in the ICBM 152 reconstruction, each with the estimated cortical current density values, only those located within a 5 mm distance from the coordinates of each ROI were selected. The source signal of each ROI was then obtained by applying the principal component analysis to the source signals of all nodes in each ROI.

### Functional connectivity and networks reconstruction

First, the source signals from 40 ROIs were evaluated for each epoch. The source signals of each ROI were decomposed into the following five frequency bands: delta (1–4 Hz), theta (4–8 Hz), alpha (8–12 Hz), beta (12–30 Hz), and gamma (30–45 Hz). This signal decomposition was accomplished by using a 6th order zero-phase Butterworth infinite impulse response (IIR) band-pass filter implemented in the MATLAB Signal Processing toolbox, with the cutoff frequencies equal to the borders of each frequency band. Then, the functional connectivity between every pair of the ROIs was evaluated by the phase-locking value (PLV) that has been widely employed to evaluate phase synchronization. The PLV between each ROI pair was evaluated by averaging PLVs of all 135 epochs for each patient. Forty ROIs were selected as areas belonging to one of the five functional networks: Fronto-Parietal Network (FPN), Salience Network (SN), Cingulo-Opercular Network (CON), Default Mode Network (DMN) and Sensory-Motor Network (SMN). Each chosen neuronal region has been uniquely assigned to only one functional network. The limitation of the analysis only to preselected ROIs was dictated by an attempt to reduce the number of included ROIs, and therefore also possible pairs of functional connections between them, and to choose neuronal areas belonging to the networks having well-known functional properties, which might be referred to cognitive models of Mind Wandering and the previous neuroimaging results. Table S1 in the Supplementary Material contains a list of the exact brain areas forming each of described five functional networks. All possible pairs of connected nodes were divided into two main groups: intra-network connections covering synchronized nodes belonging to one network (e.g. connectivity between left posterior cingulate and left frontal superior-medial cortex, both being a part of DMN) and inter-network connections covering the synchronization between nodes belonging to two different networks (e.g. connectivity between left posterior cingulate from the DMN and right frontal superior cortex from the FPN). All ROIs’ localizations and their appurtenance to the specific functional networks were determined according to Power et al.^61^ and Thatcher et al.^62^.

### Statistical analysis

The final sample of participants was divided based on the empirical median score of the MWQ into two groups. Individuals with MWQ score below the median has been assigned to a Low-MW group, and individuals with the MWQ outcomes above the median has been assigned to a High-MW group. Dispersion of the MWQ score was checked for compliance with the normal distribution. After establishing two groups differing with regard to the scope of dispositional Mind Wandering, their demographic characteristics, together with fluid intelligence indicators were compared with the Student t-test and Chi-squared test (χ2) regarding sex proportions. All these comparisons were supplemented with corresponding effect size indicators: Cohen’s d for Student t-test and the r for Chi-squared test. Basic statistics were computed using STATISTICA 13 software^63^.

The main part of analysis consisted in verifying whether there were significant differences in the organization of selected resting-state functional networks in Low-MW and High-MW groups. Having in mind that FC outcomes represent a large dataset (40 × 40 ROIs = 780 unique PLV results × 5 frequencies = 3900 values), in the current study the statistical permutation test was applied allowing for the correction for multiple comparisons and thus the adjustment of the significance *p* level. To reduce the number of PLV indexes taken into account jointly in a given comparison, all pairs of ROIs between which FC strength was established have been divided into two sets. The first one contained PLV values regarding nodes classified as belonging to one functional network (intra-network connectivity), and the second set contained PLV values regarding nodes classified as belonging to two different networks (inter-network connectivity). Here we implemented permutation test being a part of the Resampling Statistical Toolkit^58^ making 20,000 permutations of group membership to empirically approximate the distribution for the lack of inter-group hypothesis (the so-called null hypothesis), for each contrast. For every analyzed juxtaposition, a permutation was performed, on the basis of which F/t values were derived, and all F/t values for the original data that exceeded the significance threshold for the F/t distribution were considered credible^64^. Additionally, *p* values were corrected for multiple comparisons at the threshold of 0.05 using the FDR (false discovery rate) method^65^. After establishing a set of PLV values significantly differentiating subgroups Low-MW and High-MW, the analysis of the relationship between MW and FC was supplemented with the evaluation of the linear correlation (Pearson's r) between the total score in MWQ and selected PLV outcomes in the whole research group.

## Supplementary Information

Supplementary Table S1.

## Data Availability

The datasets generated during and analysed during the current study are available from the corresponding author on reasonable request.
