# Supplementary material for: Relationships between resting-state EEG functional networks organization and individual differences in mind wandering
Source: Sci Rep. 2022 Dec 8;12:21224. doi: 10.1038/s41598-022-25851-6 (PMC9731960; doi:10.1038/s41598-022-25851-6)
Supplement: Supplementary file 1 — Supplementary Table S1. [file 41598_2022_25851_MOESM1_ESM.docx]

| **Network** | **Areas** |
| --- | --- |
|  |  |
| Fronto-parietal FPN |  |
|  | frontal superior cortex L |
|  | frontal superior cortex R |
|  | frontal middle cortex L |
|  | frontal middle cortex R |
|  | parietal superior cortex L |
|  | parietal superior cortex R |
| Salience SN |  |
|  | insula L |
|  | insula R |
|  | anterior cingulate L |
|  | anterior cingulate R |
|  | amygdala L |
|  | amygdala R |
| Cingulo-opercular CON |  |
|  | middle cingulate L |
|  | middle cingulate R |
|  | frontal inferior operculum L |
|  | frontal inferior operculum R |
|  | Rolandic operculum L |
|  | Rolandic operculum R |
|  | thalamus L |
|  | thalamus R |
| Default-mode DMN |  |
|  | posterior cingulate L |
|  | posterior cingulate R |
|  | precuneus L |
|  | precuneus R |
|  | inferior parietal cortex L |
|  | inferior parietal cortex R |
|  | middle temporal cortex L |
|  | middle temporal cortex R |
|  | frontal superior-medial cortex L |
|  | frontal superior-medial cortex R |
|  | frontal orbital-medial cortex L |
|  | frontal orbital-medial cortex R |
|  | parahippocampal L |
|  | parahippocampal R |
| Sensory-Motor SMN |  |
|  | precentral L |
|  | precentral R |
|  | postcentral L |
|  | postcentral R |
|  | paracentral lobule L |
|  | paracentral lobule R |

**Supplementary material**

**Table S1.** A set of neural structures belonging to the analyzed functional networks.
